# Tumour resection and coronary artery bypass grafting for right ventricular fibroma with severe calcification and coronary artery stenosis

**DOI:** 10.1093/ehjimp/qyae076

**Published:** 2024-07-19

**Authors:** Yoshito Ito, Takahide Yoshio, Masao Daimon, Shinsuke Aida, Shuichiro Takanashi

**Affiliations:** Department of Cardiac Surgery, International University of Health and Welfare, Mita Hospital, 1-4-3 Mita, Minato-ku, Tokyo 108-8329, Japan; Department of Cardiac Surgery, International University of Health and Welfare, Mita Hospital, 1-4-3 Mita, Minato-ku, Tokyo 108-8329, Japan; Cardiology, International University of Health and Welfare, Mita Hospital, 1-4-3 Mita, Minato-ku, Tokyo, Japan; Diagnostic Pathology, International University of Health and Welfare, Mita Hospital, 1-4-3 Mita, Minato-ku, Tokyo, Japan; Department of Cardiac Surgery, International University of Health and Welfare, Mita Hospital, 1-4-3 Mita, Minato-ku, Tokyo 108-8329, Japan

**Keywords:** calcified fibroma, coronary artery disease, simultaneous surgery, cardiac magnetic resonance imaging, cardiac computed tomography, three-dimensional echocardiography

A 62-year-old female with familial hypercholesterolaemia, hypertension, and lower extremity artery disease presented with intermittent claudication. Although she had no cardiac complaints, cardiac workup was performed because she had multiple risk factors for ischaemic heart disease. Electrocardiogram showed no ischaemic signs or arrhythmia. Echocardiography showed a mobile, highly echogenic 12.7 × 7.3 mm right ventricular (RV) mass (*Figure 1A*, Supplementary data online, *Videos S1–S3*). Computed tomography (CT) and magnetic resonance imaging identified a calcified and mobile RV mass, which could be calcified amorphous tumour, calcified myxoma, or papillary fibroelastoma (*Figure 1B and C*, Supplementary data online, *Videos S4–S6*). Coronary angiography showed significant stenosis in the left main coronary artery, left anterior descending artery (LAD), left circumflex artery (LCX), and right coronary artery (RCA) (Supplementary data online, *Videos S7–S9*).

**Figure 1:**
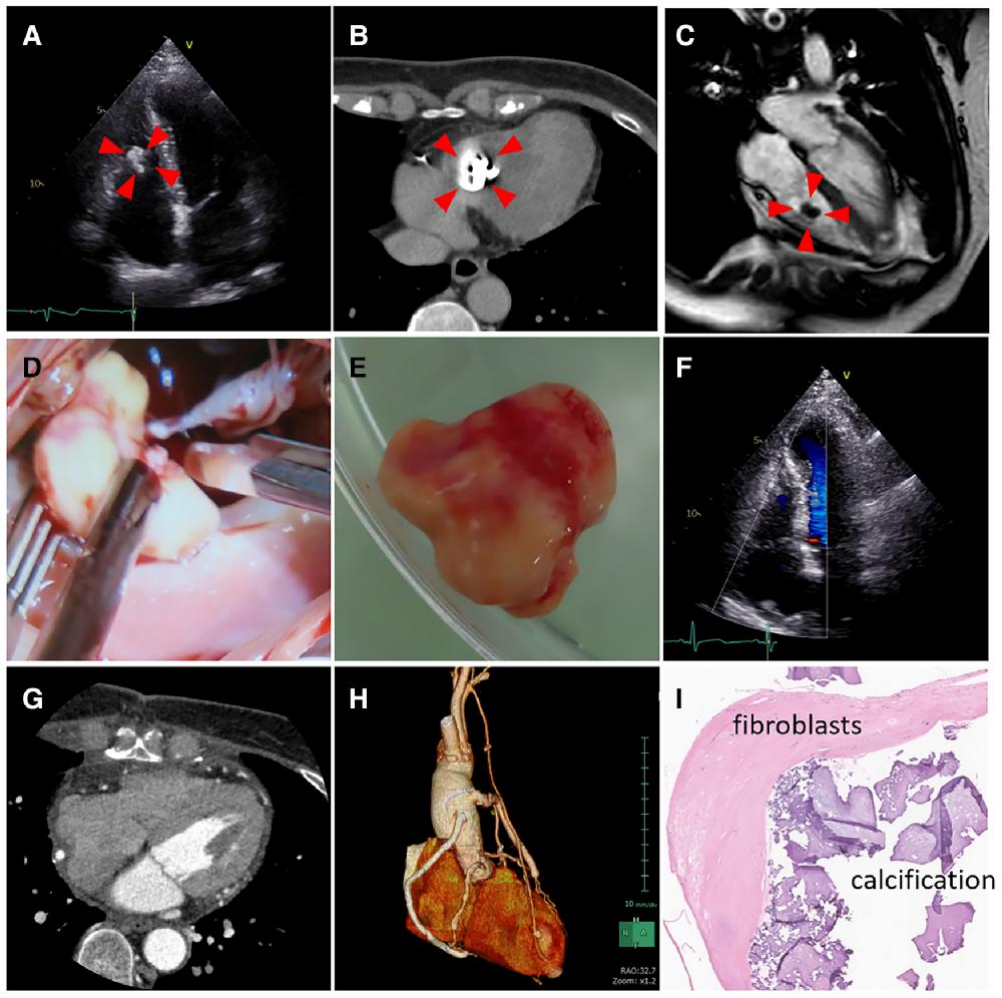
Pre-, intra-, and post-operative images and photos for this case (*A–C*) Pre-operative images of echocardiography (*A*), CT (*B*), and MRI (*C*). The red arrows shows the mass in the RV. (*D*, *E*) Intra-operative photos of the calcified mass. (*F*, *G*) Post-operative images of echocardiography (*F*), and CT (*G*). There is no residual mass and tricuspid regurgitation. (*H*) All bypass grafts are patent in three-dimensional CT. (*I*) The microscopic image with HE stain of the mass. Fibroblasts and calcification coexist, and this microscopic image confirm that the mass is a fibroma. CT, computed tomography; HE, hematoxylin Eosin; MRI, magnetic resonance imaging; RV, right ventricle.

Resection of the RV mass and coronary artery bypass grafting (CABG) to LAD, diagonal branch, LCX, and RCA was performed. The mass, with marked calcification adhering to the anterior chordae of the tricuspid valve, was excised from the RV with preserving the chordae (*Figure 1D and E*). Post-operative imaging confirmed no residual mass, no worsened tricuspid regurgitation, and all graft patency (*Figure 1F–H*, Supplementary data online, *Videos S10–S12*). Histopathological examination revealed the mass was cardiac fibroma with calcification, characterized by fibroblasts and collagen fibres on haematoxylin-eosin staining (*Figure 1I*). A follow-up CT 1-year later showed no recurrence of the tumour and the patency of the graft.

Cardiac fibromas are rare benign tumours more common in childhood and typically found in the left ventricle. Cardiac fibromas of RV in adult are rare. Additionally, the mechanism of calcification in fibromas is unclear. While simultaneous fibroma resection and CABG have not been reported previously, predisposing factors for arterial sclerosis like ageing and dyslipidaemia may contribute to fibroma calcification.

## Supplementary Material

qyae076_Supplementary_Data

## Supplementary data

**Consent**: Informed consent was given by the patient.

**Funding:** None declared.

**Data availability:** The data underlying this article will be shared on reasonable request to the corresponding author.

## Lead author biography

Yoshito Ito graduated from Osaka University in 2013, and now, he is a cardiovascular surgeon at the International University of Health and Welfare Mita Hospital, Japan. He was also engaged in basic research on regenerative medicine as a post-graduate student at Osaka University. His main interests are coronary heart disease, valvular heart disease, cardiomyopathy, and aortic disease.

